# Modulating Uranium Binding Affinity in Engineered Calmodulin EF-Hand Peptides: Effect of Phosphorylation

**DOI:** 10.1371/journal.pone.0041922

**Published:** 2012-08-03

**Authors:** Romain Pardoux, Sandrine Sauge-Merle, David Lemaire, Pascale Delangle, Luc Guilloreau, Jean-Marc Adriano, Catherine Berthomieu

**Affiliations:** 1 CEA, DSV IBEB, Laboratoire des Interactions Protéine-Métal, Saint-Paul-lez-Durance, France; 2 CNRS, UMR Biologie Végétale et Microbiologie Environnementale, Saint-Paul-lez-Durance, France; 3 Université d’Aix-Marseille, Saint-Paul-lez-Durance, France; 4 CEA, INAC, Service de Chimie Inorganique et Biologique (UMR_E 3 CEA UJF), Grenoble, France; 5 CEA, DSV IBEB, Laboratoire de Bioénergétique et Biotechnologie des Bactéries et Microalgues, Saint Paul-lez-Durance, France; Medical School of Hannover, United States of America

## Abstract

To improve our understanding of uranium toxicity, the determinants of uranyl affinity in proteins must be better characterized. In this work, we analyzed the contribution of a phosphoryl group on uranium binding affinity in a protein binding site, using the site 1 EF-hand motif of calmodulin. The recombinant domain 1 of calmodulin from *A. thaliana* was engineered to impair metal binding at site 2 and was used as a structured template. Threonine at position 9 of the loop was phosphorylated *in vitro*, using the recombinant catalytic subunit of protein kinase CK2. Hence, the T_9_TKE_12_ sequence was substituted by the CK2 recognition sequence TAAE. A tyrosine was introduced at position 7, so that uranyl and calcium binding affinities could be determined by following tyrosine fluorescence. Phosphorylation was characterized by ESI-MS spectrometry, and the phosphorylated peptide was purified to homogeneity using ion-exchange chromatography. The binding constants for uranyl were determined by competition experiments with iminodiacetate. At pH 6, phosphorylation increased the affinity for uranyl by a factor of ∼5, from K_d_ = 25±6 nM to K_d_ = 5±1 nM. The phosphorylated peptide exhibited a much larger affinity at pH 7, with a dissociation constant in the subnanomolar range (K_d_ = 0.25±0.06 nM). FTIR analyses showed that the phosphothreonine side chain is partly protonated at pH 6, while it is fully deprotonated at pH 7. Moreover, formation of the uranyl-peptide complex at pH 7 resulted in significant frequency shifts of the ν_as_(P-O) and ν_s_(P-O) IR modes of phosphothreonine, supporting its direct interaction with uranyl. Accordingly, a bathochromic shift in ν_as_(UO_2_)^2+^ vibration (from 923 cm^−1^ to 908 cm^−1^) was observed upon uranyl coordination to the phosphorylated peptide. Together, our data demonstrate that the phosphoryl group plays a determining role in uranyl binding affinity to proteins at physiological pH.

## Introduction

Uranium is a radioactive heavy metal, which is naturally present in varying concentrations in the environment. However, the wide use of uranium for industrial and military applications increases the risk of its distribution in the environment, which is aggravated by such factors as mining activities, uranium processing, or leaching of radioactive wastes.

Uranium presents radiological and chemical toxicity to living organisms [1], [2]. In spite of an increasing number of publications in recent years, information regarding the specific molecular interactions involved in uranyl chemical toxicity *in vivo* remains limited. The published data concerning the mechanism of uranium interaction with proteins at the molecular level is limited [3]–[10] and few quantitative studies have investigated the binding properties of uranyl with proteins or peptides [9], [11]–[17].

It is thus of great interest to better characterize these interactions, and to analyze structural factors governing uranyl binding and thermodynamic stabilization in proteins. Research in this direction will benefit our understanding of the molecular factors governing uranyl toxicity and speciation in cells and will also aid in developing new molecules for selectively binding uranium that could be used for uranium biodetection or bioremediation purposes [18]–[23].

In biological media, uranium is predominantly found in its hexavalent oxidation state U(VI) as the linear dioxo uranyl form (UO_2_^2+^). Uranyl forms preferred coordination to five or six hard acid donor ligands in the equatorial plane. In proteins, these ligands may be provided by oxygen atoms from carbonyl, carboxylate, phenolate, or phosphoryl groups [6], [24]. Analysis of average uranium – ligand bond distances in uranyl organic complexes has shown that phenolate and phosphoryl groups exhibit the shortest average distances to uranium [24], suggesting that these groups have high affinities for uranyl. In fact, a tripodal derivative bearing *gem*-bis-phosphonate moieties was observed to demonstrate the highest complexation properties with uranyl in a screen of molecules developed for decorporation [25]. The affinity of uranyl for phosphate groups is also exemplified by the formation of uranyl-phosphate minerals such as meta-autunite, or by uranyl efficient binding to phospholipids [26], [27], and to phosphorylated proteins such as phosvitin [28] or the S-layer proteins of *Bacillus sphaericus* JG-A12 isolated from a uranium mining waste pile [4], [19]. However, there has been no quantitative analysis of the effect that adding a phosphoryl group has on uranyl affinity, in uranium binding sites of proteins. To address this issue, we have analyzed the effect of introducing a phosphoryl group in the calcium binding loop of the calmodulin EF-hand motif on uranyl binding affinity.

Uranyl coordination properties have similarities with those of calcium. The two metal cations both form electrostatic interactions preferentially with hard donor oxygen ligands, and the preferred Ca^2+^ coordination geometry -seven ligands arranged in distorted octahedral or pentagonal bipyramidal structures- is similar to that of uranium in uranyl complexes. Recently, it was also shown that uranyl can compete with the binding of Ca^2+^ to albumin or to the C reactive protein [10], [15]. Calcium binding proteins were also evidenced among uranium binding proteins from kidney cells through uranyl-affinity chromatography *in vitro* [29].

**Figure 1:**
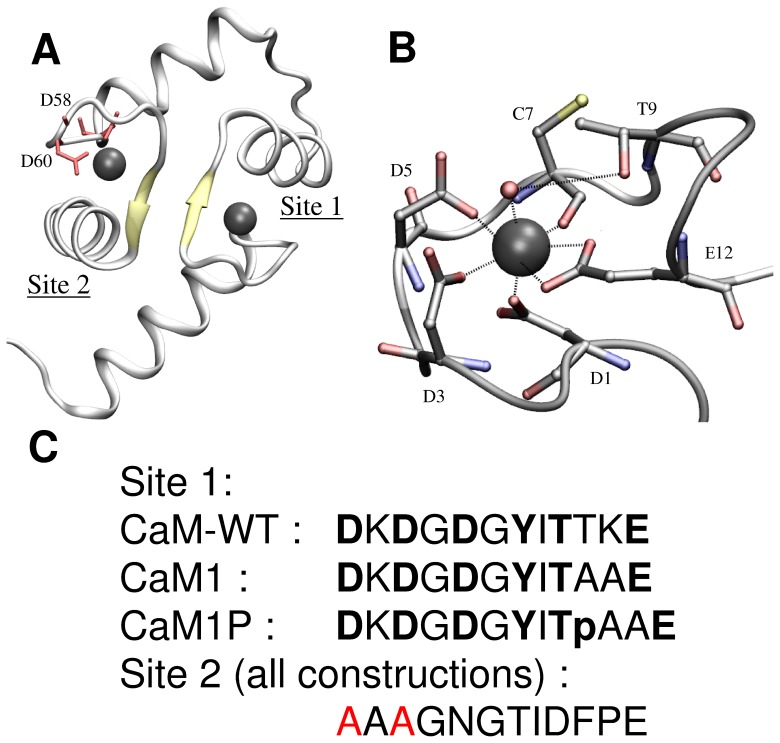
Schematic of calmodulin of *Paramecium tetraurelia*, (1EXR). A) Schematic of the calmodulin domain 1. The aspartate residues that appear in red in site 2 have been mutated into alanine for the three peptides analyzed in this study; B) Schematic of the calcium binding site 1 of *Paramecium tetraurelia* calmodulin; C) sequences of the corresponding site 1 and site 2 in the peptides analysed in this study (D corresponds to aspartate, T to threonine, L to leucine, F to phenylalanine, K to lysine, G to glycine, Y to tyrosine, I to isoleucine, and E to glutamate.).

The EF-hand structural motif is the most prevalent Ca^2+^-binding site in proteins, and is also among the five most common motifs in animal cells [30]. It is structured by two orthogonal α-helices that flank a flexible metal binding loop composed of 12 highly conserved residues, which provides the coordinating residues at positions 1, 3, 5, 7, 9, and 12. Calmodulin is the most studied representative of the ubiquitous EF-hand protein family. In site 1, calcium ligands are provided by three monodentate aspartate at positions 1, 3, 5, a bidentate glutamate at position 12, a main chain carbonyl at position 7, and a water molecule stabilized by threonine 9 side chain, as schematized in Figure 1 [31].

This structured but flexible motif providing a metal coordination sphere with hard acid ligands makes it very appealing to analyze uranyl binding properties and to develop affine and specific uranyl binding sites. Moreover, it was shown that uranyl binds with an apparent dissociation constant in the micromolar range to a 33-amino acids cyclic peptide corresponding to the helix-loop-helix calcium binding site 1 of calmodulin [13].

For these reasons, we selected one of the EF-hand motifs of calmodulin from *Arabidopsis thaliana* to analyze its uranyl binding properties. Using *in vitro* phosphorylation of threonine 9, we measured how adding a phosphoryl group affects the calcium and uranium binding affinities. We showed that the affinity for uranyl largely increases upon phosphorylation, notably at physiological pH (pH 7), wherein the phosphothreonine side chain is deprotonated, and that the phosphoryl group is involved in uranyl coordination.

## Results

### Design and Characterization of a Phosphorylated Variant of the EF-hand Binding Motif

Calmodulin is a calcium binding protein involved in the regulation of a wide range of target enzymes [32]. It contains two pairs of EF-hand motifs in two domains separated by a flexible α-helix [31]. We analyzed calcium and uranyl binding properties for site 1 variants in *Arabidopsis thaliana* calmodulin. This was accomplished by using the recombinant domain one of calmodulin, corresponding to a 77 amino acids sequence, in which the metal binding ability of site 2 was impaired by introducing Asp58Ala and Asp60Ala point mutations (Figure 1). The sequence coding for domain 1 was further modified to introduce a tyrosine at position 7 in the metal binding loop of site 1 (mutation Cys28Tyr, with numbering according to the recombinant peptide sequence). This enabled the determination of metal binding isotherms by following tyrosine fluorescence emission at 302 nm.

The recombinant peptides were produced in *E. coli*, as detailed in the Methods. A histidine-tag followed by the Tobacco Etch Virus protease (TEV) recognition sequence was introduced at the N-terminus, allowing the purification of the peptides using two subsequent chromatography steps on Ni-columns.

Phosphorylation of threonine at position 9 of the metal binding loop (Thr30 in the recombinant peptide) was performed enzymatically *in vitro* using the recombinant catalytic α subunit of protein kinase CK2 [33]. Only the α subunit of CK2 was used, and not the holoenzyme consisting of both α and β subunits, since it was shown for calmodulin that the regulatory β subunit plays a negative role on calmodulin phosphorylation [34].

The CK2 consensus recognition sequence is T(S)XXE/D/pS/pY [35]. The presence of negatively charged side chains surrounding the target amino acid is very important for CK2 activity. The crucial position is n+3, and 90% of the consensus sequences contain an acidic determinant at this position [36]. This position is occupied by a glutamate in the native calmodulin sequence. Conversely, positively charged residues at n+1 or n+2 positions strongly decrease CK2 efficiency [36]. Therefore, a variant, was constructed (referred to hereafter as CaM1) to obtain an efficient CK2 consensus sequence that targets phosphorylation of Thr at position 9 of the metal binding loop (Thr30 in the peptide). The native sequence T_30_TKE was replaced by substituting Thr31 and Lys32 each with an alanine residue, thus generating the TAAE sequence. We chose to substitute Thr31 to avoid any possible phosphorylation of this threonine.

**Figure 2:**
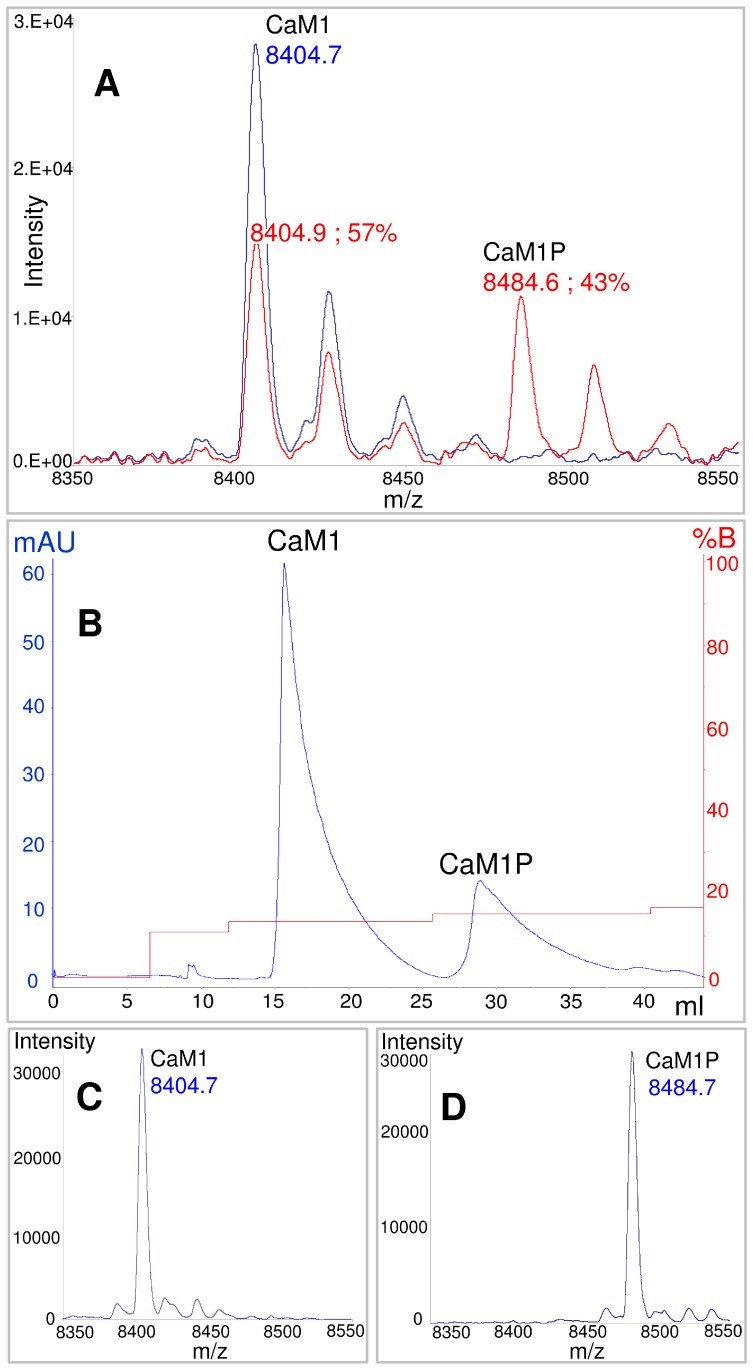
ESI-MS analysis and separation of CaM1 and CaM1P peptides. A) ESI-MS spectra recorded before (blue line) and 24 h after (red line) the phosphorylation treatment; B) elution profile of the ion-exchange chromatography; C) ESI-MS spectrum of the first purified fraction, corresponding to the non-phosphorylated CaM1 peptide; D) ESI-MS spectrum of the second purified fraction, corresponding to the phosphorylated CaM1P peptide.

*In vitro* phosphorylation was performed following a previously published method for qualitative phosphorylation using radiolabeled phosphate [37]. In addition, to improve the amount of phosphorylation, several parameters were optimized, including the substrate/enzyme ratio, the concentration in substrate, ATP and poly-L-lysine, temperature and incubating time. Phosphorylation levels were monitored by ESI-MS spectrometry, and values from 30 to 40% were obtained upon 24 h incubation with the CK2 reaction mix (Figure 2A). Purification of the phosphorylated fraction was achieved first by a thorough suppression of ATP from the sample using a desalting column, and then by separation using strong anion exchange chromatography, as detailed in the Methods (Figure 2B). The homogeneity and purity of the phosphorylated fraction was verified by ESI-MS. The purified fraction corresponded to 100% of phosphorylated peptide, referred to as CaM1P (Figure 2D).

To identify the phosphorylation site(s), trypsin digestions of purified fractions of the unphosphorylated and phosphorylated CaM1 and CaM1P peptides were analyzed by ESI-MS. The CaM1 peptide map obtained after trypsin digestion is presented in Table 1. Four or five proteolytic fragments were generated and assigned by ESI-MS with a 98.7% sequence coverage. Only the final lysine of the sequence was missing. Trypsin digestion of the phosphorylated CaM1P peptide resulted in the formation of five peptides. Only two of these peptides, 16–39 and 24–39, were identified with a mass corresponding to one phosphorylation. These peptides present the threonine of the targeted phosphorylation site (T_30_AAE). These results show clearly that we phosphorylated the targeted threonine.

**Table 1:** Amino-acid sequence of CaM1 and CaM1P, indicating the recovered trypsin-cleaved fragments.

Calmodulinpeptides map	[S_1_MADQLTDDQISEF_15_ ] [(K_16_EAFSLFDK_23_ ) (D_24_GDGYITAAELGTVMR_39_ ) ] [S_40_LGQNPTEAELQDMINEVAAAGNGTIDFPEFLNLMAR_76_ ] K	
*m/z* values	Peptide and mass	Sequence
CaM1		
864.39^2+^; 1727.78^+^	1–15 : 1726.8 Da	SMADQLTDDQISEFK(E)
869.08^3+^; 1303.62^2+^	16–39 : 2605.2 Da	(K)EAFSLFDKDGDGYITAAELGTVMR(S)
1326.64^3+^; 1989.95^2+^	40–76 : 3976.9 Da	(R)SLGQNPTEAELQDMINEVAAAGNGTIDFPEFLNLMAR(K)
CaM1P		
864.39^2+^; 1727.78^+^	1–15 : 1726.8 Da	SMADQLTDDQISEFK(E)
896.07^3+^; 1343.6^2+^	16–39 : 2685.2 Da	(K)EAFSLFDKDGDGYI**Tp**AAELGTVMR(S)
956.46^+^	16–23 : 955.4 Da	(K)EAFSLFDK(D)
874.87^2+^	24–39 : 1747.7 Da	(K)DGDGYI**Tp**AAELGTVMR(S)
995.22^4+^; 1989.44^2+^; 1326.6^3+^	40–76 : 3976.9 Da	(R)SLGQNPTEAELQDMINEVAAAGNGTIDFPEFLNLMAR(K)

Parentheses and brackets in the sequence delimit the assigned proteolytic fragments.

Summary of identified peptides for non-phosphorylated CaM1 and phosphorylated CaM1P. Phosphorylated threonine is labeled in bold.

### Binding Affinities for Uranyl and Calcium

The effect of threonine phosphorylation on uranium and calcium binding affinities was measured by fluorescence titrations on CaM-WT, CaM1, and CaM1P at pH 6 and pH 7.

For titrations with uranyl, iminodiacetate (IDA) was added to the peptide solution to control uranyl speciation and to avoid the formation of hydroxo uranyl complexes, which are formed at pH greater than 4. Indeed, IDA has been demonstrated to be a uranyl ligand and the stability constants of its uranyl complexes have been measured by potentiometry at 25°C with a ionic strength of 0.1 M (KNO_3_) [38].

**Figure 3:**
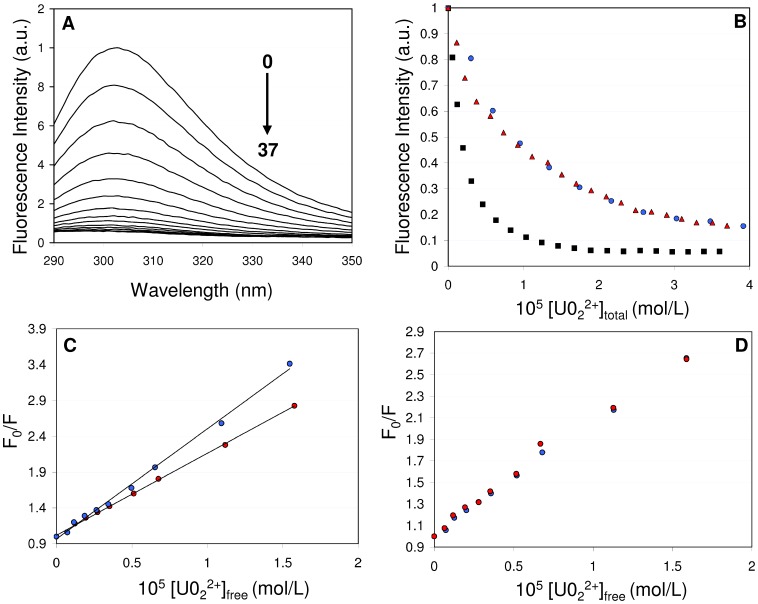
Fluorescence analysis of the CaM1 and CaM1P peptides. A) Fluorescence spectra of the CaM-WT peptide (10 µM in 10 mM MES buffer, pH6) in the presence of increasing concentrations (0 to 37 µM) of added uranyl nitrate. B) Maximum emission spectra (at 302 nm) of CaM-WT (blue), CaM1 (red) and CaM1P (black) as a function of added uranium concentration. C) Stern-Volmer representations for CaM1 at 15°C (blue) or 35°C (red); the lines correspond to the stern-Vomer fit. D) Stern-Volmer representation for CaM1 in the absence (blue) or presence (red) of 0.1 M glycerol.

The peptides were prepared at a 10 µM concentration in 20 mM MES pH 6 or Tris pH 7, with 0.1 M KCl and 100 µM IDA. Increasing concentrations of uranyl nitrate were added to the peptide solution, until the peptide to uranyl ratio was approximately 1∶4. By using this stoichiometric ratio, the protein samples were not affected by uranyl addition (as monitored by UV-Vis absorption), which is crucial for the interpretation of the results. Addition of uranyl nitrate decreased the fluorescence signal emitted by the single tyrosine present in the peptides at position 7 of the metal binding loop (Figure 3A). Tyrosine fluorescence quenching by uranyl has been reported in the literature for other proteins such as transferrin [16]. No shift in the Tyr emission maximum at 302 nm was observed after addition of uranyl nitrate to the solution, indicating that the peptide-uranyl interaction did not alter the tyrosine local micro-environment. Figure 3B depicts the evolution of tyrosine fluorescence as a function of additive uranyl nitrate concentrations for the three samples CaM-WT, CaM1 and CaM1P at pH 6, under the conditions detailed in the Methods.

Fluorescence quenching can result from a variety of molecular interactions, including especially static or dynamic quenching. Therefore, for each protein studied, we investigated the nature of the fluorescence quenching. Stern-Volmer representations are presented in Figures 3C and D for the peptide CaM1. The Stern-Volmer representation plots the ratio F_0_/F versus [UO_2_]_free_. In our experiment, the Stern-Volmer plots are linear, which proves that the quenching is either purely dynamic or static [39]. Static and dynamic quenching can be distinguished by their differing dependence on viscosity, temperature or by lifetime measurements. In our case, higher temperatures decreased the value of the slope of the Stern Volmer plots, as shown for CaM1 in Figure 3C. This proves that the fluorescence quenching originates from a static phenomenon [39]. Moreover, no changes were observed upon increasing the sample viscosity by addition of glycerol (at 0.1 M, Figure 3D), which is also consistent with a purely static quenching.

For each peptide studied, dissociations constants of the peptide – uranyl complexes (K_d_ in equation 1) were determined at pH 6 and pH 7 (Table 2). These conditional constants result from the competition experiments with IDA and were calculated by fitting the fluorescence emission spectra of tyrosine and taking into account the three uranyl complexes formed with IDA (UO2IDA, [UO2(IDA)2]^2−^ and [(UO2)2(IDA)2(OH)2]^2−^) according to equations 2–4, where P stands for peptide.


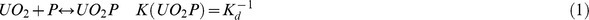



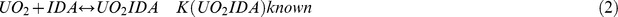



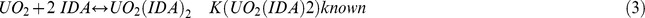



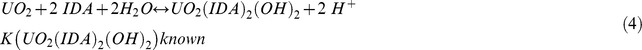


Therefore, the conditional stability constants of the three uranyl complexes formed with IDA (UO_2_IDA, [UO_2_(IDA)_2_]^2−^ and [(UO_2_)_2_(IDA)_2_(OH)_2_]^2−^) were calculated at pH 6 and pH 7 from the pK_a_s of IDA and the stability constants of these complexes [38]. Their values were fixed in the analysis of the titrations of the peptides with uranyl done in presence of 10 equivalents of IDA. These competition experiments confirmed the formation of 1∶1 complexes of uranyl with the three peptides and allowed us to determine the uranyl binding affinities.

**Table 2:** Uranyl and calcium dissociation constants (K_d_) for the 1∶1 complexes of the peptides with uranyl and calcium.

	K_d_ (nM) for uranyl		K_d_ (µM) for calcium	
Peptides	pH6	pH7	pH6	pH7
CaM-WT	32±7		38.4±1.01 (R = 0.9994)	27.2±1.2 (R = 0.9978)
CaM1	25±6		23.2±2.69 (R = 0.9911)	22.4±1.13 (R = 0.9982)
CaM1P	5±1	0.32±0.057	21.5±1.47 (R = 0.9959)	17.1±1.57 (R = 0.9912)

*R is the correlation coefficient.*

The mutations designed to allow phosphorylation of the threonine at position 9 of the metal binding loop by CK2 (Thr31Ala and Lys32Ala) do not significantly affect the peptide-uranyl interaction properties, as revealed by the conditional dissociation constants obtained for CaM-WT (K_d_ = 32±7 nM) and CaM1 (K_d_ = 25±6 nM) at pH 6. Threonine phosphorylation increased the peptide affinity for uranyl by a factor of ∼5 at pH 6, with a dissociation constant of 5±1 nM for the CaM1P–uranyl complex (Table 2). Compared to pH 6, the binding affinity of CaM1P for uranyl was improved by more than a factor of 15 at pH 7, and the dissociation constant of the CaM1P-uranyl complex lied in the subnanomolar range with K_d_ = 0.32±0.06 nM (Table 2). These data demonstrate that phosphorylation has a large effect on the affinity of the peptide for uranyl at physiological pH.

Tyrosine fluorescence was also used to determine conditional dissociation constants of the different peptides with calcium (Table 2). Addition of calcium enhanced Tyr fluorescence as previously described in the literature (Supporting Information S1 [40]). Conditional dissociation constants in the micromolar range were obtained when CaM-WT and CaM1 (at 10 µM concentration) were titrated with CaCl_2_ in MES 20 mM pH 6, 0.1 M KCl. Almost no effect of pH was observed on the dissociation constants calculated for the CaM-WT and CaM1 peptides (ranging from K_d_ = 38.4 µM at pH 6 to K_d_ = 22.4 µM at pH 7, Table 2). The effect of threonine phosphorylation on the affinity for calcium was negligible at pH 6, and very modest at pH 7, which elicited an increase by a factor of 1.3 (Table 2).

### Analysis of the Phosphoryl Group Using FTIR Spectroscopy

The significant increase in affinity for uranyl evidenced with the phosphorylated peptides suggests a direct involvement of phosphothreonine in uranyl coordination. Moreover, the effect of pH on CaM1P affinity for uranyl may be due in part to the pK_a_ of phosphothreonine, which has been reported at 5.9–6.1 in phosphorylated peptides [41], [42]. We thus used Fourier transform infrared (FTIR) spectroscopy to investigate the protonation state of the phosphothreonine side-chain in CaM1P and its possible involvement in uranyl coordination. The results are summarized in Table 3.

**Table 3:** Summary and proposed assignment of the main infrared bands observed in the 1350–750 cm^−1^ region.

Proposed assignments	CaM1 pH 6	CaM1 pH 7	CaM1P pH 6		CaM1P pH 7	
IR spectra	CaM1U –*minus*- CaM1	CaM1U –*minus*- CaM1	CaM1P –*minus*-CaM1	CaM1P-U –*minus*-CaM1	CaM1P –*minus*-CaM1	CaM1P-U –*minus*-CaM1
Protein contributions	1287	1292		1288		1294
Protein contributions	1225	1260		1221		1262
ν(P = O) (−OPO_3_H^−^)			1215	1179		
ν_as_(P-O) (−OPO_3_H^−^)			1178	1151		
ν_s_(P-O) (−OPO_3_H^−^)			1090			
ν_as_(P-O) (−OPO_3_^2−^)			1066	1064	1068	1064
ν_s_(P-O) (−OPO_3_^2−^)				978	967	978
ν_as_(P-OH) (−OPO_3_H)			954			
ν_3_(UO_2_^2+^)	920	915		913		908

**Figure 4:**
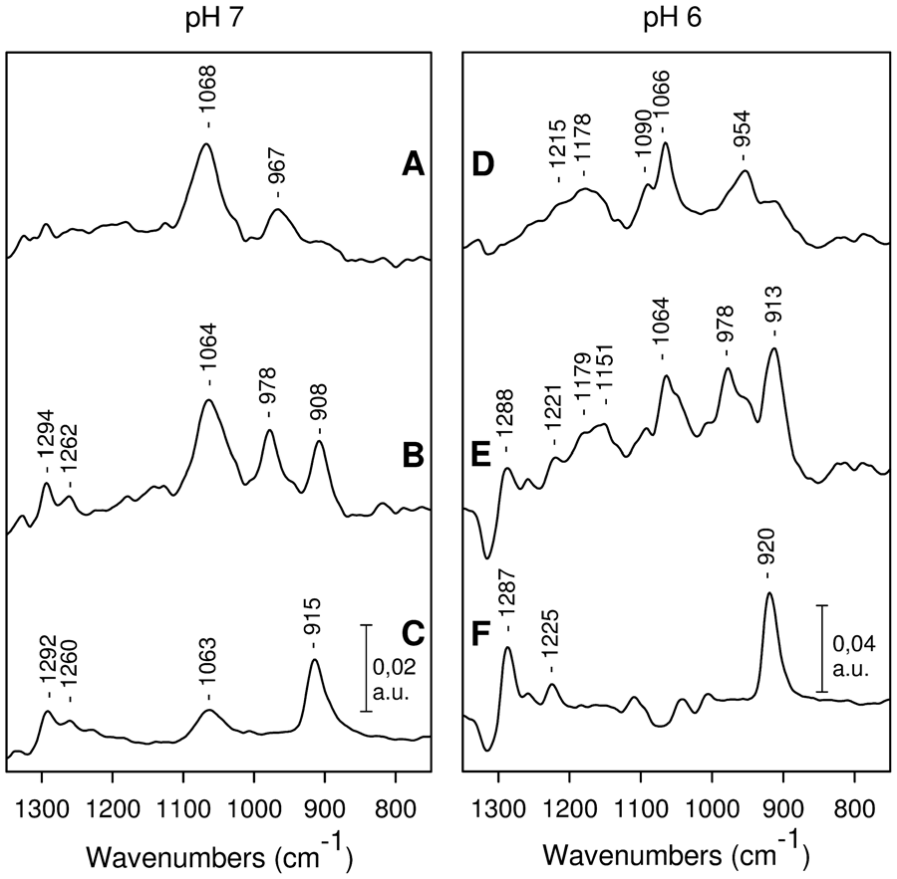
FTIR difference spectra recorded with the CaM peptides. CaM1P-*minus*-CaM1 at pH 7 (A) and at pH 6 (D), CaM1P-U-*minus*-CaM1 at pH 7 (B) and at pH 6 (E), CaM1-U-*minus*-CaM1 at pH 7 (C) and at pH 6 (F). The difference spectra were obtained as detailed in the Methods.

Infrared modes of the phosphoryl group of phosphothreonine are expected to contribute in the 1300–750 cm^−1^ range [28], [43], [44]. Other IR contributions from the peptide may also be observed in this spectral region. Therefore, to selectively probe the IR modes of phosphothreonine in CaM1P, we subtracted the absorption spectrum of the corresponding unphosphorylated peptide CaM1 from the absorption spectrum of the phosphorylated peptide CaM1P. Only IR modes of the phosphothreonine side chain are expected to contribute to the resulting difference spectra CaM1P-*minus*-CaM1 (see Figure 4A for pH 7 and Figure 4D for pH 6).

Two bands were observed at 1068 and 967 cm^−1^ in the CaM1P-*minus*-CaM1 spectrum recorded at pH 7 (Figure 4A). These bands were assigned to the antisymmetric ν_as_(P-O) and symmetric ν_s_(P-O) stretching modes of the di-anionic −OPO_3_^2−^ group of phosphothreonine, based on prior designations in the literature [27], [28]. The ν(C-OP) mode may also contribute in part to the band at 1068 cm^−1^ [43], [44].

In the CaM1P-*minus*-CaM1 spectrum recorded at pH 6, the ν_as_(P-O) mode of the di-anionic −OPO_3_^2−^ group was observed at 1066 cm^−1^ (Figure 4D). Additional bands were observed at 1215, 1178, 1090 and 954 cm^−1^ in this spectrum. These bands are assigned to the ν(PO), ν_as_(P-O), ν_s_(P-O) and ν_s_(P-OH) modes of the protonated −OPO_3_H^−^ group of phosphothreonine based on literature data [27], [28]. Thus, the FTIR data reveal that phosphothreonine is predominantly in its monoanionic (−OPO_3_H^−^) form at pH 6, while it is fully deprotonated as the di-anionic species (−OPO_3_^2−^) in CaM1P at pH 7.

The IR modes of phosphothreonine were analyzed in the CaM1P-uranyl complex (CaM1P-U), by recording difference spectra between the absorption of the complex CaM1P-U minus the absorption of the unphosphorylated peptide CaM1 without uranyl (CaM1P-U-*minus*-CaM1). These spectra are displayed in Figures 4B (at pH 7) and 4E (at pH 6). The complex was formed with a peptide : uranyl ratio of one, as described in the Methods. IR modes from the phosphoryl group and from uranyl are expected to contribute to these spectra. The difference spectra between the absorption of the complex CaM1-U minus the absorption of CaM1 were also recorded for comparison (see Figures 4C for pH 7 and 4F for pH 6). In these spectra the main band at 915 cm^−1^ (pH 7) or 920 cm^−1^ (pH 6) is due to uranyl (see below). Other bands observed at 1292 and 1260 cm^−1^ at pH 7 (Figure 4C) or at 1287 cm^−1^ and 1225 cm^−1^ at pH 6 (Figure 4F) are due to protein IR modes of the CaM1-U complex. These bands were observed at almost the same frequencies in the CaM1P-U-*minu*s-CaM1 spectra (Figures 4C and 4E). Assignment of these bands is not straightforward. The presence of these bands in both CaM1-U-*minus*-CaM1 and CaM1P-U-*minus*-CaM1 spectra indicates however that they correspond to similar structural changes and/or common residues involved in uranyl coordination in the CaM1-U and CaM1P-U complexes.

In the CaM1P-U complex at pH 7, the ν_as_(P-O) and ν_s_(P-O) modes of phosphothreonine are shifted by −4 and +11 cm^−1^, respectively, to 1064 cm^−1^ and 978 cm^−1^ (Figure 4B; note that there is a small band at 1063 cm^−1^ in the CaM1-U-*minus*-CaM1 spectrum, and that this band may contribute in part to the 1064 cm^−1^ band observed in Figure 4B). A change in the splitting of the ν_as_ and ν_s_(P-O) modes of organic phosphate is associated with its direct interaction with uranyl [28]. Moreover, the ν_s_(P-O) mode is expected to be more sensitive to geometrical changes in the −OPO_3_^2−^ moiety than the ν_as_(P-O) mode [45]. The IR data presented in Figure 4 thus clearly indicate that the phosphoryl group of threonine is involved in uranium coordination at pH 7.

Bands at 1064 and 978 cm^−1^ were also observed at pH 6 (Figure 4E). This indicates that part of the CaM1P-U complex formed at pH 6 involves an interaction between the dianionic phosphoryl group of phosphothreonine and uranyl. Additional bands were observed in the spectrum recorded at pH 6, at 1179–1151 cm^−1^ and at 954 cm^−1^. These bands correspond to IR modes of the monoanionic species −OPO_3_H^−^, showing that a fraction of phosphothreonine remains protonated in the presence of uranyl at pH 6. Therefore, part of the CaM1P-U complexes formed at pH 6 may involve protonated threonine.

A large band was observed in the CaM1P-U complexes, either at 908 cm^−1^ (pH 7) or at 913 cm^−1^ (pH 6). It corresponds to the ν_3_(UO_2_^2+^) asymmetric stretching mode of uranyl. In the CaM1-U complex, this band was observed at 915 cm^−1^ (pH 7) or at 920 cm^−1^ (pH 6, Figures 4C and 4F). Thus, a downward shift in the uranyl ν_3_(UO_2_^2+^) IR mode by 7 cm^−1^ is observed in the CaM1P-U complexes.

The ν_3_(UO_2_^2+^) mode frequency is sensitive to the nature and number of uranyl equatorial ligands [46], [47], [48], [49]. The fully hydrated uranyl ion shows a band at 961 cm^−1^ [48], [50], whereas the frequency for carboxylate complexes of uranyl has been reported at 930–918 cm^−1^ [49], and even as low as 870 cm^−1^ for uranyl complexes involving four hydroxyl ligands [47]. Bands at 918–905 cm^−1^ were observed for uranyl-phosvitin and -lipopolysaccharides complexes involving phosphoryl groups [4], [27], [28]. In particular, there was a contribution from the ν_3_(UO_2_^2+^) mode at 918 cm^−1^ in uranyl-lipopolysaccharide complexes involving deprotonated phosphoryl groups [27]. From this literature data, we conclude that the lower ν_3_(UO_2_^2+^) mode frequencies observed for the CaM1P-U complexes (as compared to the CaM1-U complexes) strongly support a direct coordination of uranyl by the phosphothreonine phosphoryl group in CaM1P-U.

## Discussion

To analyze the effect of phosphorylation on uranyl binding affinity in a protein binding site, we investigated uranyl binding properties at the metal binding loop of the EF-hand motif of calmodulin site 1 (Figure 1), using the recombinant domain 1 of calmodulin from *A. thaliana*. We used the entire domain 1 to benefit from a structured and stable protein template, which allowed us to engineer point mutations in the metal binding loop without promoting large structural changes. It was also shown previously that metal binding did not affect the structure of domain 1 [51].

Moreover, threonine at position 9 of the EF-hand metal binding loop (Thr30 in the peptide) is ideally suited for analyzing the effect of phosphorylation on uranyl and calcium binding affinities. Its side chain is oriented towards calcium, with its hydroxyl oxygen atom located 5.39 Å from the calcium, and it stabilizes a water molecule directly coordinated to the calcium cation (Figure 1). We thus anticipated a possible contribution of the phosphoryl group from phosphothreonine in uranyl coordination. In addition, this threonine is located three amino acids upstream of a glutamate residue involved in calcium coordination. This enabled us to insert the recognition sequence of the protein kinase CK2–TAAE- without large changes to the metal binding sequence, and high yields of specific peptide phosphorylation were obtained by optimising the *in vitro* reaction.

Binding of uranyl to the unphosphorylated CaM-WT and CaM1 peptides was characterized by conditional dissociation constants in the nanomolar range (K_d_ = 32±7 and 25±6 nM, respectively). By comparison, a binding constant of the same order (K_d_ = 53 nM) was reported for the uranyl binding site engineered from the Ni binding site of the transcriptional repressor NikR [9], using a similar competition approach. Uranyl is expected to be coordinated by the side chains of two aspartates and two histidines at this site, while in the metal binding loop of calmodulin site 1, uranyl ligands may be provided by oxygen atoms from aspartate and/or peptide carbonyl groups. Although we cannot directly compare our results with those obtained by other measuring approaches, it is interesting to note that dissociation constants in the 10^−9^ M to 10^−10^ M range were reported for the interaction of monoclonal antibodies with uranyl complexed to dicarboxylic-phenanthroline derivatives [18], [22].

Phosphorylation of the threonine at position 9 of the metal binding loop increased uranyl binding affinity by a factor of 5 at pH 6 in CaM1P, while increasing the pH to 7 led to a further enhancement in uranyl affinity by a factor of 15.6. Thus, at pH 7, the dissociation constant obtained for the uranyl complex with CaM1P is in the subnanomolar range, with a K_d_ of 3 10^−10^ M.

To identify the molecular origin of the large effect of pH on the uranyl binding affinity of CaM1P, we analyzed the properties of the phosphothreonine side chain in the peptide using FTIR spectroscopy. Our results indicate that the effect of pH on uranyl affinity is due, at least in part, to the pK_a_ of the phosphothreonine side chain. Indeed, only partial deprotonation of the phosphoryl group was observed in CaM1P at pH 6, in accordance with a pK_a_ of ≈6, which is a typical value reported for phosphothreonine in the literature [41], [42]. On the contrary, the phosphoryl group is fully deprotonated in the dianionic form −OPO_3_^2−^ at pH 7.

In the CaM1P-U complex formed at pH 7, the frequencies of the IR modes of the phosphothreonine side chain and of uranyl strongly support a direct involvement of the deprotonated phosphoryl group in uranyl coordination. At pH 6, a fraction of the monoanionic phosphoryl group is still detected in the CaM1P-U complex. At this pH, two types of CaM1P-uranyl complexes may exist in solution, with the major form involving a weak electrostatic interaction of the protonated phosphorylated side chain of phosphothreonine with uranyl. Therefore, the pK_a_ of phosphothreonine and the prevalence of the latter CaM1P-uranyl complex at pH 6 are responsible for the small decrease in K_d_ observed at pH 6 upon CaM1 phosphorylation. In contrast, at pH 7, the dianionic phosphoryl group is in direct strong interaction with uranyl leading to a more stable complex and a strong decrease in K_d_.

Collectively, these results show that the affinity of the EF-hand motif for uranyl increases by almost two orders of magnitude upon phosphorylation of Thr9, at pH 7, i.e. when the phosphoryl group is in the di-anionic form. Although a conformational change associated with the binding of uranyl to CaM1P at pH 7 may also contribute to a decrease in K_d_, our data suggest a determinant role for phosphorylated protein sites in uranium chelation in cells, associated with phosphoryl deprotonation at physiological pH. These data thus underline the possible role of phosphorylated proteins in determining uranyl speciation in cells. Among strong uranyl ligands, the phosphoryl groups present the advantage over phenolates of having a pK_a_ in the physiological pH range, which explains their potential impact on uranyl binding in biological systems.

Interestingly, we observed that phosphorylation of CaM1 only has a modest impact on its affinity for calcium. Similar dissociation constants were obtained for the CaM1-Ca and CaM1P-Ca complexes at pH 6 (K_d_ ∼23 µM), and only a small increase in affinity was observed for the CaM1P-Ca complex at pH 7, probably due to the increased negative charge of the di-anionic phosphoryl group. Peptides with phosphorylated binding sites are thus very interesting to develop new high affinity uranyl ligands, which in addition could provide a good selectivity for this toxic metal ion with respect to calcium.

The necessity for new specific metal biosensors or chelators for cost-effective uranium bioremediation strategies will only become more important. Our combined approach of phosphorylation and amino acid substitution at the metal binding loop of the EF-hand motif can address this need, with its relative ease of developing potentially useful peptide-derived affine and specific uranyl binding sites. Future direction of this work will involve the structural analysis of the peptide-uranyl complexes to correlate structural properties and uranium binding affinity in the phosphorylated peptides.

## Methods

### Plasmids, Bacterial Strains and Growth Conditions

#### Engineering and purification of calmodulin derived

The CaM-D1 construct containing the *Arabidopsis thaliana* sequence of calmodulin domain 1, was obtained as previously described [51], and used as a template for new constructs. In this sequence, a tyrosine was introduced at position 7 of the metal binding loop [51]. All mutations were produced with the QuickChange site-directed mutagenesis kit (Stratagene) and specific primer pairs (see Supporting Information S1), according to the manufacturer’s instructions. Asp58Ala and Asp60Ala mutations were produced in both CaM-WT and CaM1 constructs to inactivate the metal binding site 2 of domain 1. In addition, Thr31Ala and Lys32Ala mutations were produced in the CaM1 construct. Subsequently, each mutated DNA sample was amplified with the D1-CaM-TEV-S and D1-CaM-STOP-AS primers (see Supporting Information S1) to introduce the TEV protease recognition site upstream of the coding sequence. PCR amplification was performed using 2.5 U of Taq polymerase (Prime Star; Lonza) for 30 cycles with 250 ng of linearized template DNA under the following cycling conditions : denaturation at 98°C for 10 s, hybridization at 55°C for 10 s and polymerization at 72°C for 20 s. DNA fragments (282 bp) containing each coding sequence were isolated after digestion with *BamH*I and *Pst*I, sequenced and ligated into the *BamH*I and *Pst*I sites of plasmid pQE30 to obtain the PQE-CaM-WT and PQE-CaM1 plasmids.

Recombinant fusion proteins expressed in *E. coli* strain M15Rep4 (Qiagen) were grown at 37°C in LB medium containing ampicillin (50 μg/mL) and kanamycin (50 μg/mL). Expression was induced by addition of 0.1 mM isopropyl-D-thiogalactoside once OD_600_ reached 0.5, and the cultures were further incubated for 5 h at 37°C. Cellular extracts were obtained by French press lysis and a centrifugation step of 30 min at 15000 rpm, and were applied at a 1 mL/min flow rate on a 5 mL HiTrap Chelating Column (GE Healthcare) in buffer A (50 mM Tris-HCl, 0.5 M NaCl, 25 mM imidazole buffer pH 7.5) containing 1 mM AEBSF. The proteins were eluted from the nickel resin at a 4 mL/min flow rate using buffer A supplemented with 150 mM imidazole. The proteins were dialyzed against buffer A and the His-Tags were removed by incubation overnight at 4°C with TEV protease, followed by separation using a HiTrap Chelating Column. Recombinant proteins were dialyzed against 50 mM Tris-HCl, 150 mM NaCl, pH 7.5. The protein concentrations were measured according to the BC Assay (Uptima) with bovine serum albumin as standard. The proteins were concentrated using the Microcon filtration system (Amicon Millipore®), with a cut-off point of 3 kDa.

#### Plasmid constructs, expression and purification of αCK2

Plasmid pT7 containing the human cDNA encoding the α subunit of CK2, kindly provided by Dr. Lorenzo Pinna (University of Padova), was used as a template for PCR amplification of αCK2. Primers were expressly designed to introduce a *BamH*I restriction site upstream of the initiator codon and a *Pst*I restriction site downstream of the stop codon (Supporting Information S1). PCR amplification was performed using 2.5 U of Taq polymerase (Prime Star; Lonza) for 30 cycles with 250 ng of linearized template DNA under the following cycling conditions : denaturation at 98°C for 10 s, hybridization at 55°C for 10 s and polymerization at 72°C for 72 s. A 1200 bp DNA fragment containing the αCK2 coding sequence was isolated after digestion with *BamH*I and *Pst*I, sequenced and ligated into the *BamH*I and *Pst*I sites of plasmid pQE30 to obtain the pQE-αCK2 plasmid. The latter was then introduced into *E. coli* strain M15Rep4.

Recombinant fusion proteins were expressed under the same conditions as those described for the CaM peptides (CaM1 and CaM-WT), with the exception that induction was performed overnight at 25°C. αCK2 purification was performed as described for the CaM peptides. The αCK2 proteins were concentrated using the Microcon filtration system (Amicon Millipore®) with a cut-off point of 10 kDa.

#### In vitro phosphorylation of the CaM peptides

*In vitro* phosphorylation assays were performed by preincubating 50 µM of CaM peptides for 10 min in a reaction mixture containing: 50 mM Tris-Cl pH 7, 2 mM ATP, 10 mM MgCl_2_, 1 µM poly-L-Lysine and 1 mM EGTA. The reaction was then initiated by addition of 0.5 µM of αCK2 and performed at 37°C for 24 h. Phosphorylated CaM peptides were desalted with a Hiprep desalting column (GE Healthcare) and loaded onto a MonoQ column 5/50 GL (GE Healthcare) in 20 mM MES pH 5.5. Phosphorylated peptides were eluted with the MES buffer containing 200 mM NaCl.

All protein samples were then electrophoresed on a 12% Tris-Tricine gel and stained with Coomassie Blue.

### Mass Spectrometry Analyses

Mass spectrometry (MS) analyses were performed on a MicroTOF-Q Bruker (Wissembourg, France) with an electrospray ionization source (ESI). CaM peptides were diluted in CH_3_CN/H_2_O (1/1-v/v), 0.2% formic Acid. Samples were continuously infused at a 3 µL/min flow rate. Mass spectra were recorded in the 300–3000 mass-to-charge (*m/z*) range. MS experiments were carried out with a capillary voltage set at 4.5 kV and an end plate off set voltage of 500 V. The gas nebulizer (N_2_) pressure was set at 0.4 bars and the dry gas flow (N_2_) at 4 L/min at 190°C.

Data were acquired in the positive mode and calibration was performed using a calibrating solution of ESI Tune Mix (Agilent) in CH_3_CN/H_2_O (95/5-v/v). The system was controlled by the software package MicroTOF Control 2.2 and data were processed with DataAnalysis 3.4.

### Trypsin Digestion

Trypsin (Promega) solution (0.2 µg/µL) was prepared with Promega buffer. CaM1 and CaM1P peptide digestions were performed at 37°C for 6 hours in NH_4_HCO_3_ 50 mM at pH 7.8 with a protease:substrate ratio of 1∶20 (w/w).

### Tyrosine Fluorescence Titrations

The metal-binding affinity of the various peptides for calcium and uranyl was examined by monitoring the fluorescence intensity of the single tyrosine residue (Tyr28).

The uranyl solutions were prepared extemporaneously by diluting a 0.1 M stock solution of uranyl nitrate (pH 3.5, stored frozen at −20°C) in the final buffer. For fluorescence titrations in the presence of uranyl, we used a 10 µM peptide solution in MES (20 mM pH 6) or Tris (20 mM pH 7) buffer with 100 mM KCl and 100 µM IDA. For fluorescence titrations in the presence of calcium, we used in a 10 µM peptide solution in MES (20 mM pH 6) or Tris (20 mM pH 7) buffer with 100 mM KCl. To remove any trace of calcium from the samples, each sample solution was incubated 1 h with Chelex-100 before uranyl or calcium addition.

Spectra were collected on a Cary eclipse spectrofluorimeter at 25°C, with 270 nm excitation. Emission was observed from 290 to 350 nm. The excitation and emission slits were 10 nm. A 15 min equilibration time was respected before each measurement. The reported stability constants are averages of three experimental values.

For all titrations with uranyl nitrate, the nature of the fluorescence quenching was investigated using the Stern Volmer equation given by:


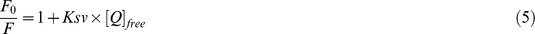


where F_0_ and F are the fluorescence intensities in the absence and presence of quencher, *Ksv* is the Stern-Volmer constant and [Q]_free_ is the free uranyl concentration. [Q]_free_ was calculated by the following equation:


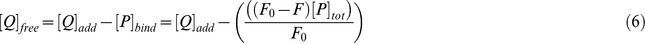


in which [Q]_add_ is the concentration of added quencher uranyl and [P]_bind_ and [P]_tot_ correspond to the concentration of bound peptide and the total peptide concentration, respectively. In the case where the complexed species is non-fluorescent and the nature of quenching is static, the Stern-Volmer constant *Ksv* is the reverse of the dissociation constant Kd.

Competition experiments between CaM peptides and IDA were performed to determine the conditional dissociation constants of the peptide-uranyl complexes at pH 6 and pH 7. IDA has a moderate affinity for uranyl and forms three major complexes: UO_2_IDA, [UO_2_(IDA)_2_]^2−^, and [(UO_2_)_2_(IDA)_2_(OH)_2_]^2−^. The conditional stability constants of these three species were calculated from the pK_as_ and the stability constants at 25°C and 0.1 M ionic strength given by Jiang et al. [38]. These three conditional stability constants were fixed in the spectral data analysis, which was performed using the program SPECFIT [52]. Identical values were obtained for the conditional stability constants of the UO_2_-P complexes (where P stands for peptide), either considering that the UO_2_-P complex emits or not. In the former case, the spectrum of the UO_2_-P complex was calculated to be zero, as the fluorescence emission of tyrosine was totally quenched in the complex.

For titrations in the presence of calcium, the conditional dissociation constants (K_d_) were determined by fitting the difference between fluorescence intensities measured in the presence (F) and in the absence (F0) of calcium, according to a one site saturation model : ΔF = (F_max_ x [Ca])/(Kd + [Ca]) using SigmaPlot 10.0 software (Systat Software, Point Richmond, CA). In this equation, F_max_ corresponds to the maximum of fluorescence determined by the software.

### FTIR Spectroscopy

The FTIR spectra were recorded on a Bruker IFS28 FTIR spectrometer equipped with a DTGS detector. We used an Attenuated Total Reflection (ATR) device (*SensIR* Technologies, CT) fitted with a 9 bounce diamond microprism with a 4.3 mm surface diameter and ZnSe optics. Each single beam spectrum corresponded to 300 co-added scans at 4 cm^−1^ resolution. All frequencies reported have an accuracy of ±1 cm^−1^. Spectra correspond to the average of data recorded with two samples.

For sample preparation, 500 µL of a 50 µM peptide solution was prepared through successive concentrations and dilutions in MES 200 µM pH 6 or Tris 200 µM pH 7 buffers. Each buffer was treated with Chelex-100 to remove any traces of calcium. The uranyl stock solution was made by diluting the 0.1 N UO_2_(NO_3_)_2_ stock solution. The uranyl solution was added dropwise to obtain a 1∶1 peptide:U ratio. The samples were then concentrated to 2 mM peptide concentration and washed by dilution in the buffer without uranium before a final concentration using a Microcon filtration system (Amicon Millipore^®^) with a cut-off point of 3 kDa. 5 µL volumes of 2 mM peptide solutions were deposited on the diamond crystal and dried. Under these conditions, the sample absorption was greater than 1 at frequencies greater than 1550 cm^−1^, and the difference spectra were not accurate in this frequency domain. Therefore, interpretation of the FTIR data was restricted to the 1450–900 cm^−1^ region. Absorption spectra of the peptides were obtained after subtracting the absorption spectra of the buffers, recorded in the same conditions.

## Supporting Information

Supporting Information S1 Supporting information contains Table S1 including the primers used to generate the peptide variants and Figure S1 including the binding thermograms of CaM peptides with calcium at pH 6 and pH 7.
(PDF)

## References

[1] Taylor DM, Taylor SK (1997) Environmental uranium and human health. Rev Environ Health 12: 147–157. 9406286 10.1515/reveh.1997.12.3.147

[2] Brugge D, de Lemos JL, Oldmixon B (2005) Exposure pathways and health effects associated with chemical and radiological toxicity of natural uranium: a review. Rev Environ Health 20: 177–193. 16342416 10.1515/reveh.2005.20.3.177

[3] Ananyev GM, Murphy A, Abe Y, Dismukes GC (1999) Remarkable affinity and selectivity for Cs+ and uranyl (UO22+) binding to the manganese site of the apo-water oxidation complex of photosystem II. Biochemistry 38: 7200–7209. 10353831 10.1021/bi990023u

[4] Merroun ML, Raff J, Rossberg A, Hennig C, Reich T, et al (2005) Complexation of uranium by cells and S-layer sheets of Bacillus sphaericus JG-A12. Appl Environ Microbiol 71: 5532–5543. 16151146 10.1128/AEM.71.9.5532-5543.2005 PMC1214696

[5] Ansoborlo E, Prat O, Moisy P, Den Auwer C, Guilbaud P, et al (2006) Actinide speciation in relation to biological processes. Biochimie 88: 1605–1618. 16996675 10.1016/j.biochi.2006.06.011

[6] Van Horn JD, Huang H (2006) Uranium(VI) bio-coordination chemistry from biochemical, solution and protein structural data. Coordination Chemistry Reviews 250: 765–775.

[7] Vidaud C, Gourion-Arsiquaud S, Rollin-Genetet F, Torne-Celer C, Plantevin S, et al (2007) Structural consequences of binding of UO_2_(2+) to apotransferrin: can this protein account for entry of uranium into human cells? Biochemistry 46: 2215–2226. 17266333 10.1021/bi061945h

[8] Hartsock WJ, Cohen JD, Segal DJ (2007) Uranyl acetate as a direct inhibitor of DNA-binding proteins. Chemical Research in Toxicology 20: 784–789. 17432879 10.1021/tx600347k

[9] Wegner SV, Boyaci H, Chen H, Jensen MP, He C (2009) Engineering A Uranyl-Specific Binding Protein from NikR. Angewandte Chemie-International Edition 48: 2339–2341. 19199314 10.1002/anie.200805262

[10] Pible O, Vidaud C, Plantevin S, Pellequer JL, Quemeneur E (2010) Predicting the disruption by UO(2)(2+) of a protein-ligand interaction. Protein Science 19: 2219–2230. 20842713 10.1002/pro.501 PMC3005792

[11] Scapolan S (1998) Uranium (VI)-Transferrin System Studied by Time-Resolved Laser-Induced Fluorescence. Radiat Prot Dosim 79: 505–508.

[12] Huang H, Chaudhary S, Van Horn JD (2005) Uranyl-peptide interactions in carbonate solution with DAHK and derivatives. Inorg Chem 44: 813–815. 15859249 10.1021/ic049528l

[13] Le Clainche L, Vita C (2006) Selective binding of uranyl cation by a novel calmodulin peptide. Environmental Chemistry Letters 4: 45–49.

[14] Duff MR, Kumar CV (2006) Site-selective photocleavage of proteins by uranyl ions. Angewandte Chemie-International Edition 45: 137–139. 10.1002/anie.200502344 16299822

[15] Montavon G, Apostolidis C, Bruchertseifer F, Repinc U, Morgenstern A (2009) Spectroscopic study of the interaction of U(VI) with transferrin and albumin for speciation of U(VI) under blood serum conditions. Journal of Inorganic Biochemistry 103: 1609–1616. 19800129 10.1016/j.jinorgbio.2009.08.010

[16] Michon J, Frelon S, Garnier C, Coppin F (2010) Determinations of uranium(VI) binding properties with some metalloproteins (transferrin, albumin, metallothionein and ferritin) by fluorescence quenching. J Fluoresc 20: 581–590. 20066480 10.1007/s10895-009-0587-3

[17] Averseng O, Hagege A, Taran F, Vidaud C (2010) Surface plasmon resonance for rapid screening of uranyl affine proteins. Anal Chem 82: 9797–9802. 21069968 10.1021/ac102578y

[18] Blake RC, Pavlov AR, Khosraviani M, Ensley HE, Kiefer GE, et al (2004) Novel monoclonal antibodies with specificity for chelated uranium(VI): Isolation and binding properties. Bioconjugate Chemistry 15: 1125–1136. 15366969 10.1021/bc049889p

[19] Pollmann K, Raff J, Merroun M, Fahmy K, Selenska-Pobell S (2006) Metal binding by bacteria from uranium mining waste piles and its technological applications. Biotechnology Advances 24: 58–68. 16005595 10.1016/j.biotechadv.2005.06.002

[20] Liu JW, Brown AK, Meng XL, Cropek DM, Istok JD, et al (2007) A catalytic beacon sensor for uranium with parts-per-trillion sensitivity and millionfold selectivity. Proceedings of the National Academy of Sciences of the United States of America 104: 2056–2061. 17284609 10.1073/pnas.0607875104 PMC1892917

[21] Hillson NJ, Hu P, Andersen GL, Shapiro L (2007) Caulobacter crescentus as a whole-cell uranium biosensor. Applied and Environmental Microbiology 73: 7615–7621. 17905881 10.1128/AEM.01566-07 PMC2168040

[22] Reisser-Rubrecht L, Torne-Celer C, Renier W, Averseng O, Plantevin S, et al (2008) High-affinity uranyl-specific antibodies suitable for cellular imaging. Chemical Research in Toxicology 21: 349–357. 18154273 10.1021/tx700215e

[23] Melton SJ, Yu H, Williams KH, Morris SA, Long PE, et al (2009) Field-Based Detection and Monitoring of Uranium in Contaminated Groundwater using Two Immunosensors. Environmental Science & Technology 43: 6703–6709. 19764238 10.1021/es9007239

[24] Pible O, Guilbaud P, Pellequer JL, Vidaud C, Quemeneur E (2006) Structural insights into protein-uranyl interaction: towards an in silico detection method. Biochimie 88: 1631–1638. 16815621 10.1016/j.biochi.2006.05.015

[25] Sawicki M, Siaugue JM, Jacopin C, Moulin C, Bailly T, et al (2005) Discovery of powerful uranyl ligands from efficient synthesis and screening. Chemistry-a European Journal 11: 3689–3697. 10.1002/chem.200401056 15809989

[26] Koban A, Bernhard G (2007) Uranium(VI) complexes with phospholipid model compounds - A laser spectroscopic study. Journal of Inorganic Biochemistry 101: 750–757. 17320184 10.1016/j.jinorgbio.2007.01.001

[27] Barkleit A, Foerstendorf H, Li B, Rossberg A, Moll H, et al (2011) Coordination of uranium(VI) with functional groups of bacterial lipopolysaccharide studied by EXAFS and FT-IR spectroscopy. Dalton Trans 40: 9868–9876. 21879077 10.1039/c1dt10546a

[28] Li B, Raff J, Barkleit A, Bernhard G, Foerstendorf H (2010) Complexation of U(VI) with highly phosphorylated protein, phosvitin A vibrational spectroscopic approach. Journal of Inorganic Biochemistry 104: 718–725. 20385410 10.1016/j.jinorgbio.2010.03.004

[29] Dedieu A, Berenguer F, Basset C, Prat O, Quemeneur E, et al (2009) Identification of uranyl binding proteins from human kidney-2 cell extracts by immobilized uranyl affinity chromatography and mass spectrometry. Journal of Chromatography A 1216: 5365–5376. 19501829 10.1016/j.chroma.2009.05.023

[30] Ye YM, Lee HW, Yang W, Shealy S, Yang JJ (2005) Probing site-specific calmodulin calcium and lanthanide affinity by grafting. Journal of the American Chemical Society 127: 3743–3750. 15771508 10.1021/ja042786x

[31] Babu YS, Sack JS, Greenhough TJ, Bugg CE, Means AR, et al (1985) Three-dimensional structure of calmodulin. Nature 315: 37–40. 3990807 10.1038/315037a0

[32] Chin D, Means AR (2000) Calmodulin: a prototypical calcium sensor. Trends in Cell Biology 10: 322–328. 10884684 10.1016/s0962-8924(00)01800-6

[33] Pinna LA (2002) Protein kinase CK2: a challenge to canons. J Cell Sci 115: 3873–3878. 12244125 10.1242/jcs.00074

[34] Marin O, Meggio F, Sarno S, Pinna LA (1997) Physical dissection of the structural elements responsible for regulatory properties and intersubunit interactions of protein kinase CK2 beta-subunit. Biochemistry 36: 7192–7198. 9188720 10.1021/bi962885q

[35] Marchiori F, Meggio F, Marin O, Borin G, Calderan A, et al (1988) Synthetic peptide substrates for casein kinase 2. Assessment of minimum structural requirements for phosphorylation. Biochim Biophys Acta 971: 332–338. 3167103 10.1016/0167-4889(88)90149-8

[36] Meggio F, Pinna LA (2003) One-thousand-and-one substrates of protein kinase CK2? FASEB J 17: 349–368. 12631575 10.1096/fj.02-0473rev

[37] Quadroni M, L’Hostis EL, Corti C, Myagkikh I, Durussel I, et al (1998) Phosphorylation of calmodulin alters its potency as an activator of target enzymes. Biochemistry 37: 6523–6532. 9572870 10.1021/bi972930+

[38] Jiang J, Renshaw JC, Sarsfield MJ, Livens FR, Collison D, et al (2003) Solution chemistry of uranyl ion with iminodiacetate and oxydiacetate: A combined NMR/EXAFS and potentiometry/calorimetry study. Inorg Chem 42: 1233–1240. 12588161 10.1021/ic020460o

[39] Lakowicz JR (2006) Principles of fluorescence spectroscopy. Springer Science & Business Media, LCC, New York 3rd Ed..

[40] VanScyoc WS, Sorensen BR, Rusinova E, Laws WR, Ross JBA, et al (2002) Calcium binding to calmodulin mutants monitored by domain-specific intrinsic phenylalanine and tyrosine fluorescence. Biophysical Journal 83: 2767–2780. 12414709 10.1016/S0006-3495(02)75286-7 PMC1302361

[41] Hoffmann R, Reichert I, Wachs WO, Zeppezauer M, Kalbitzer HR (1994) 1H and 31P NMR spectroscopy of phosphorylated model peptides. Int J Pept Protein Res 44: 193–198. 7529751 10.1111/j.1399-3011.1994.tb00160.x

[42] Xie Y, Jiang Y, Ben-Amotz D (2005) Detection of amino acid and peptide phosphate protonation using Raman spectroscopy. Analytical Biochemistry 343: 223–230. 16018962 10.1016/j.ab.2005.05.038

[43] Correia CF, Balaj PO, Scuderi D, Maitre P, Ohanessian G (2008) Vibrational signatures of protonated, phosphorylated amino acids in the gas phase. Journal of the American Chemical Society 130: 3359–3370. 18293967 10.1021/ja073868z

[44] Scuderi D, Correia CF, Balaj OP, Ohanessian G, Lemaire J, et al (2009) Structural characterization by IRMPD spectroscopy and DFT calculations of deprotonated phosphorylated amino acids in the gas phase. Chemphyschem 10: 1630–1641. 19347918 10.1002/cphc.200800856

[45] Deng H, Wang JH, Callender R, Ray WJ (1998) Relationship between bond stretching frequencies and internal bonding for [O-16(4)]- and [O-18(4)]phosphates in aqueous solution. Journal of Physical Chemistry B 102: 3617–3623.

[46] Mcglynn SP, Neely WC, Smith JK (1961) Electronic Structure, Spectra, and Magnetic Properties of Oxycations.3. Ligation Effects on Infrared Spectrum of Uranyl Ion. Journal of Chemical Physics 35: 105–116.

[47] Muller K, Brendler V, Foerstendorf H (2008) Aqueous uranium(VI) hydrolysis species characterized by attenuated total reflection Fourier-transform infrared spectroscopy. Inorg Chem 47: 10127–10134. 18831578 10.1021/ic8005103

[48] Muller K, Foerstendorf H, Tsushima S, Brendler V, Bernhard G (2009) Direct Spectroscopic Characterization of Aqueous Actinyl(VI) Species: A Comparative Study of Np and U. Journal of Physical Chemistry A. 113: 6626–6632. 10.1021/jp9008948 19514785

[49] Kakihana M, Nagumo T, Okamoto M, Kakihana H (1987) Coordination Structures for Uranyl Carboxylate Complexes in Aqueous-Solution Studied by Ir and C-13 Nmr-Spectra. Journal of Physical Chemistry 91: 6128–6136.

[50] Quiles F, Burneau A (2000) Infrared and Raman spectra of uranyl(VI) oxo-hydroxo complexes in acid aqueous solutions: a chemometric study. Vibrational Spectroscopy 23: 231–241.

[51] Le Clainche L, Plancque G, Amekraz B, Moulin C, Pradines-Lecomte C, et al (2003) Engineering new metal specificity in EF-hand peptides. Journal of Biological Inorganic Chemistry 8: 334–340. 12589569 10.1007/s00775-002-0419-2

[52] Binstead RA, Zuberbühler AD, Jung B (2003) Specfit Global Analysis System Version 3.0.34.

