# Supplementary material for: Modulating Uranium Binding Affinity in Engineered Calmodulin EF-Hand Peptides: Effect of Phosphorylation
Source: PLoS One. 2012 Aug 3;7(8):e41922. doi: 10.1371/journal.pone.0041922 (PMC3411679; doi:10.1371/journal.pone.0041922)

Supplementary Table S1: Primers used to generate the peptide variants. The primers were synthesized according to *E. coli* codon usage.

Primer pairs	Sequences (5'→3')	Goal
D/Ax2 S	GGTAACGGTACCATTGATTTCCCAG	Mutations of Asp58 to Ala and Asp60 to Ala
D/Ax2 AS	AGCAGCAGCCACTTCGTTGATCATG	
TAAE S	GGTGATGGTTaCATTACCgCCgcGGAGCTGGGTACTGTGATGCG	Mutations of Thr31Lys32 to AlaAla
TAAE AS	CGCATCACAGTACCCAGCTCCgcGGcGGTAATGtAACCATCACC	
D1 CaM TEV S	GAGAGGATCCgagaacctgtacttccagtccATGGCGGATCAGCTCACCGACGAT	CaM cloning into PQE30 with TEV protease recognition site
D1 CaM Stop AS	GAGAGAGAGAGAGACTGCAGTCACTTACGAGCCATAAGGTTCAAGAAC	
S-αCK2	TATggatccATGTCGGGACCCGTGCCAAGC	αCK2 cloning into PQE30
AS-αCK2	TATctgcagTTACTGCTGAGCGCCAGCGGC	

Supplementary Figure S1: Binding thermograms of CaM peptides with calcium. CaM-WT (A), CaM1 (B) and CaM1P (C) at pH 6 and CaM-WT (D), CaM1 (E) and CaM1P (F) at pH 7.

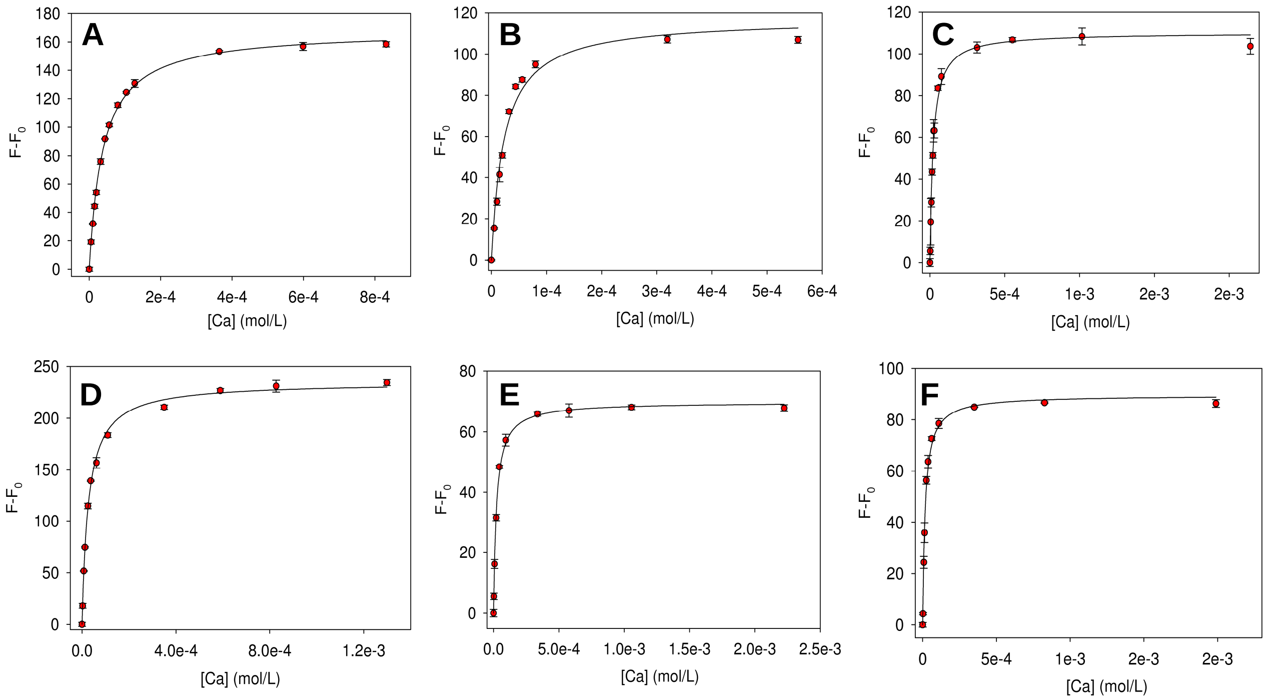

Supplement: Supporting Information S1 — Supporting information contains Table S1 including the primers used to generate the peptide variants and Figure S1 including the binding thermograms of CaM peptides with calcium at pH 6 and pH 7. (PDF) [file pone.0041922.s001.pdf]
